# Arthroscopic rotator cuff repair in fibromyalgia patients had comparable outcomes to a matched control group

**DOI:** 10.1186/s10195-023-00706-6

**Published:** 2023-05-11

**Authors:** Ron Gurel, Matias Vidra, Etay Elbaz, Shai Factor, Efi Kazum, Assaf Bivas, Eran Maman, Ofir Chechik, Dani Rotman

**Affiliations:** 1 Department of Orthopedic Surgery, Tel Aviv Sourasky Medical Center, Tel Aviv, Israel; 2 Sackler Faculty of Medicine, Tel Aviv University, Tel Aviv, Israel

**Keywords:** Fibromyalgia, Rotator cuff tear, ARCR, Arthroscopy, Shoulder

## Abstract

**Background:**

Although fibromyalgia is associated with poor outcomes following orthopedic surgeries, several studies show some benefit from surgical intervention and nevertheless recommend operative treatment when indicated. There is sparse evidence of the effect of fibromyalgia on the outcomes of shoulder surgery. The purpose of this study was to investigate the effect of fibromyalgia on patient-reported outcomes of arthroscopic rotator cuff repair (ARCR).

**Methods:**

All patients with a confirmed diagnosis of fibromyalgia who underwent ARCR in one institution between 2010 and 2021 were included. Data retrieved from medical records included demographics, characteristics of the cuff tear and the surgical procedure, and preoperative and last follow-up (minimum 1 year) postoperative Disabilities of the Arm, Shoulder and Hand (DASH) score, Subjective Shoulder score (SSV), and Numeric Pain Rating Scale (NPRS). A matched controlled group of patients without fibromyalgia who had undergone ARCR was selected according to age, sex, and preoperative DASH, SSV, and NPRS scores.

**Results:**

There were no significant differences in demographics, cuff tear and surgical procedure characteristics, and preoperative scores between the fibromyalgia and control groups. The fibromyalgia patients’ postoperative scores for all 3 measurements showed significant improvement: SSV by 32.1 (*P* = 0.004), DASH by 20.3 (*P* = 0.016), and NPRS by 2.33 (*P* = 0.017). There were no significant differences in the postoperative DASH, SSV, and NPRS between the fibromyalgia and control groups.

**Conclusion:**

Fibromyalgia patients with rotator cuff tears who undergo ARCR do not have inferior patient-reported outcomes compared with non-fibromyalgia controls. Fibromyalgia should not be a considered a contraindication for ARCR.

*Level of evidence*: III

## Introduction

Rotator cuff tears are common and 250,000 to 300,000 rotator cuff repairs (RCR) are performed in the United States every year, with an increasing percentage of them being carried out arthroscopically [1, 2]. The functional outcomes of RCR are reportedly affected by several parameters, including fear-avoidance behavior, alcohol consumption, sex, workers compensation claims, previous RCR procedure, preoperative functional score of the involved or the contralateral shoulder, smoking status, age, activities of daily living, subacromial decompression, tear size, biceps tendon intervention, preoperative pain, and muscle atrophy [3–5].

Fibromyalgia is described as a centralized pain state that is prevalent in 2–8% of the population, and characterized by widespread pain often accompanied by fatigue, memory problems, and sleep disturbances [6]. The effect of fibromyalgia on the outcomes of orthopedic surgery has been studied and reported in several publications. A recent systematic review described it as a risk factor for poor outcomes following orthopedic surgery [7]. Specific to shoulder surgery, another recent systematic review stated that due to the scarcity and low level of evidence in the available literature, it is currently impossible to determine whether fibromyalgia does or does not have an effect on shoulder surgery outcomes [8].

The aim of our study was to compare the outcomes of patients with and those without fibromyalgia who underwent arthroscopic rotator cuff repair (ARCR) in one medical institution, and to determine whether ARCR outcomes are affected by fibromyalgia. We hoped to provide clinical evidence of the effect of fibromyalgia on the outcome of surgery for rotator cuff tears to shoulder surgeons who treat patients with these coexisting pathologies.

## Materials and methods

### Study population

This was a retrospective cohort study. Following institutional ethical review board approval (no. TLV-0731-18), all consecutive adult patients treated in our rheumatology clinic due to a diagnosis of fibromyalgia and who also underwent ARCR in our institution between 2010 to 2021 were digitally located. Each case was matched according to sex, age at surgery, the preoperative Disabilities of the Arm, Shoulder and Hand (DASH) score, the Subjective Shoulder score (SSV), and the Numeric Pain Rating Scale (NPRS) with a control patient who underwent ARCR. The controls were confirmed not to have fibromyalgia. The collected data included demographics, cuff tear and surgical procedure characteristics, and the preoperative and postoperative NPRS, DASH, and SSV scores which are routinely obtained in our clinic. The patients were evaluated in our clinic preoperatively, and followed at routine postoperative visits at 2, 6, and 12 weeks, and at 6 and 12 months thereafter. Minimal follow-up time for each patient was 1 year. In cases where not all scores were available, patients were reached by a phone call to complete the missing data.

### Statistical analysis

Means and standard deviations were used to describe continuous variables. Percents were used to describe categorical variables. Preoperative, cuff tear, and surgical procedure characteristics were compared between the fibromyalgia and the control groups in order to confirm that they were appropriately matched. This was performed by using the independent samples *t*-test, Mann–Whitney *U* test, chi-square, and Fisher’s exact tests. We applied a paired sample *t*-test in order to compare the preoperative to the last follow-up postoperative NPRS, DASH, and SSV scores of the fibromyalgia group. We then compared the last follow-up NPRS, DASH, and SSV scores of the fibromyalgia group with those of the matched control group by means of an independent sample *t*-test. We also compared the difference between the scores at the last follow-up with the preoperative scores among the groups, using an independent sample *t*-test. All statistical analyses were performed with IBM SPSS V24.

## Results

Eighteen consecutive patients with a confirmed diagnosis of fibromyalgia underwent ARCR at our institution during the study period. They were matched with 18 ARCR non-fibromyalgia patients who served as the control group. There were no significant group differences in age at surgery or preoperative NPRS, DASH, or SSV scores, and the female-to-male ratio was identical (Table 1). Mean and minimum follow-up times were 51.9 ± 34.6 and 12 months, respectively. Additional analysis also showed no significant differences between the groups in both cuff tear characteristics (fatty infiltration [9], retraction, time from onset to surgery, and involved tendons) or characteristics of the surgical procedure (complete/partial repair and biceps tenotomy/tenodesis) (Table 2).

**Table 1:** Group characteristics

	Fibromyalgia group (No = 18)^a^	Control group (No. = 18)^a^	*P* value
Male/female ratio	2/16	2/16	
Age at surgery, years	58.8 ± 12.2	57.4 ± 11.6	0.73
Preoperative SSV	29.3 ± 23.439	33.0 ± 23.2	0.64
Preoperative DASH	71.8 ± 22.0	73.2 ± 13.8	0.83
Preoperative NPRS	7.83 ± 1.76	7.95 ± 2.04	0.86

*SSV* subjective shoulder score, *DASH* Disabilities of the Arm, Shoulder and Hand score, *NPRS* Numerical Pain Rating Scale.

^a^All values are mean ± standard deviation.

**Table 2:** Characteristics of the cuff tears and the surgical procedures

	Fibromyalgia group	Control group	*P* value
Fatty infiltration (mean ± SD)*	1.4 ± 0.9	1.8 ± 1.3	0.49
Tear retraction (mean ± SD[mm])	9.7 ± 9.2	13.6 ± 15.2	0.41
Time from onset to surgery (mean ± SD[months])	37.5 ± 31.3	23.9 ± 35.1	0.26
Biceps tendon tenotomy (%)	66.70%	66.70%	1
Biceps tendon tenodesis (%)	11.10%	0%	0.49
Complete/partial tear (%)	66.7%/33.3%	88.9%/11.1%	0.23
Involved tendons			
Supraspinatus (% of patients)	100%	100%	
Infraspinatus (% of patients)	33.3%	33.3%	1
Subscapularis (% of patients)	38.9%	27.8%	0.48

^*^According to Goutallier et al.

There was a significant improvement in all 3 patient-reported outcome scores in the fibromyalgia group: the SSV increased from 29.3 to 61.4 (*P* = 0.004), the DASH decreased from 71.8 to 51.5 (*P* = 0.016), and the NPRS decreased from 7.83 to 5.50 (*P* = 0.017) (Table 3). There was no significant group difference in the last follow-up postoperative SSV, DASH, or NPRS scores (Table 4), or in the mean improvement in SSV, DASH, or NPRS scores (Table 5).

**Table 3:** Comparison between postoperative and preoperative scores of the fibromyalgia group

	Preoperative^a^	Postoperative^a^	*P* value
SSV	29.3 ± 23.4	61.4 ± 23.7	0.004
DASH	71.8 ± 22.0	51.5 ± 29.1	0.016
NPRS	7.83 ± 1.76	5.50 ± 3.19	0.017

*SSV* Subjective Shoulder score, *DASH* Disabilities of the Arm, Shoulder and Hand score, *NPRS* Numerical Pain Rating Scale.

^a^All values are mean ± standard deviation.

**Table 4:** Comparison of postoperative outcomes between the fibromyalgia group and the control group

	Fibromyalgia^a^	Controls^a^	*P* value
SSV	61.4 ± 23.7	47.7 ± 26.1	0.109
DASH	51.5 ± 29.1	56.0 ± 25.3	0.623
NPRS	5.50 ± 3.19	5.56 ± 2.48	0.954

*SSV* Subjective Shoulder score, *DASH* Disabilities of the Arm, Shoulder and Hand score, *NPRS* Numerical Pain Rating Scale.

^a^All values are mean ± standard deviation.

**Table 5:** Comparison of the differences between the postoperative and the preoperative scores of the fibromyalgia and control groups

	Fibromyalgia^a^	Controls^a^	*P* value
Delta SSV	32.1 ± 41.1	14.7 ± 22.8	0.126
Delta DASH	20.3 ± 32.4	17.1 ± 21.2	0.729
Delta NPRS	2.33 ± 3.76	2.39 ± 2.68	0.960

*SSV* Subjective Shoulder score, *DASH* Disabilities of the Arm, Shoulder and Hand score, *NPRS* Numerical Pain Rating Scale.

^a^All values are mean ± standard deviation.

## Discussion

This study showed no significant group difference in postoperative outcomes of ARCR between patients with and without fibromyalgia. Moreover, the fibromyalgia group reported a significant improvement between the preoperative and the last follow-up postoperative DASH, NPRS, and SSV scores. These results suggest that ARCR is a successful procedure in patients with coexisting fibromyalgia, in opposition to several reports that claimed that fibromyalgia was a risk factor for poor postoperative outcome.

D’onghia et al.’s systematic review of the results of orthopedic surgery in general among fibromyalgia patients concluded that fibromyalgia was consistently reported as a significant risk factor for lower patient satisfaction, higher pain scores, worse functional outcome, increased risk for postoperative opioid prescription, and higher rate of medical and surgical complications [7]. Sodhi et al. performed an analysis of over 300,000 patients who underwent total knee arthroplasty and concluded that fibromyalgia patients have a greater risk of developing certain surgical complications after the procedure, such as bearing wear, aseptic loosening, infection, and dislocation [10]. In contrast to those studies and similar to our current findings, Bican et al. compared 59 patients with fibromyalgia who underwent total knee arthroplasty to non-fibromyalgia controls and reported comparable improvement between the 2 groups, concluding that fibromyalgia should not be a contraindication to the procedure [11]. Lopiz et al. compared 26 fibromyalgia patients who underwent arthroscopic subacromial decompression with 20 control non-fibromyalgia patients who underwent the same procedure, and reported that fibromyalgia was a prognostic factor for poor postoperative outcome. However, those authors nevertheless recommended surgical treatment when indicated due to some clinical improvement seen in the fibromyalgia group [12]. Compagnoni et al. performed a systematic review of fibromyalgia effect on shoulder surgery and concluded that the current literature on the topic is too limited to allow the confirmation of any impact of fibromyalgia on shoulder surgery [8].

The main limitations of our study were its retrospective nature and its small size. However, it is currently the only comparative study of postoperative outcomes of ARCR in fibromyalgia patients and we were able to reach statistical significance in several outcomes. Another limitation was that we have no data regarding the rehabilitation process of the patients and their pre- or postoperative medication use.

In conclusion, this study demonstrates comparable results of ARCR for fibromyalgia patients and non-fibromyalgia controls, and provides evidence of significant patient reported improvement in fibromyalgia patients who presented with cuff tears and underwent ARCR. Our study findings suggest that fibromyalgia should not be considered as a contraindication for ARCR in patients with rotator cuff tears.

## Data Availability

Data is available upon request from the corresponding author.
